# Laboratory Robotics in Radiation Environments

**DOI:** 10.6028/jres.093.040

**Published:** 1988-06-01

**Authors:** Tony J. Beugelsdijk, Dan W. Knobeloch

**Affiliations:** Mechanical and Electronic Engineering Division Los Alamos National Laboratory Los Alamos, NM 87544

Radiation environments pose special problems for the implementation of robotic systems. A large part of the robotic effort at Los Alamos National Laboratory is spent addressing these issues and modifying commercial robotic equipment for these environments. This paper will briefly summarize some of the problems encountered and the solutions we have implemented on three laboratory robotic systems. The degree of modification increases with each system described. This succession of experiences has led us to begin the design of our own laboratory robotic arm compatible with this environment. This effort will also be described.

Problems associated with radiation have their origins in the types of radiation encountered [1]. By far the most common are alpha and beta emitting sources. For through-space radiation, these are fairly easily dealt with by simple shielding of sensitive robotic components. Contact radiation must be avoided, however, by careful control of the particulate levels within the workcell, by physical enclosure of semiconductor components, and by gas purging. Coatings must also be removed and polymeric materials must be replaced with metal wherever possible.

Gamma and neutron sources are much more difficult to deal with due to their penetrating nature. Here, successful approaches involve the considerations for alpha and beta sources as well as remoting electronics to outside the containment area. Maintainability issues also surface for these types of radiation. Where components are likely to fail, ease of replacement becomes key, especially while wearing thick, lead-lined gloves.

An initial laboratory robotic project involved the preparation of samples containing plutonium and americium prior to radiochemical counting for these elements [2]. This system was built around commercially available components acquired from the Zymark Corporation. The robot performed multiple dilutions and extractions in addition to weighing, centrifugation, and incubations for each sample. Final preparations consisted of a dried droplet (90 microliters) on a glass cover slip which is submitted for gross alpha measurement and a test tube containing two milliliters of solution for gamma counting. The results of both measurements are taken together in the final calculations for the plutonium and americium content of the original sample.

Modifications to this system were minor due to the predominance of alpha radiation and extremely low levels of americium (<100 ppm), a gamma source. Shielding from the lab environment was accomplished with a plexiglass hood enclosure built specifically to fit over the robot and its modules. A slight negative pressure is maintained within this enclosure.

A second application involved dispensing aliquots of radioactive solutions [3]. These solutions came from dissolved core samples taken from the Nevada Test Site after a test firing. A corrosion resistant version of the standard Zymark arm was chosen for this project. A special problem was posed, however, in the handling of these solutions in the volumes required (10–300 mL) without contaminating the delivery devices. A solution was found through the use of peristaltic pumps and three-way pinch valves. These components were assembled into a pump station controlled by digital signals from Zymark’s Power and Event Controller [4]. Between test Firings, the tygon tubing is replaced to avoid cross-contamination. In addition, the master solutions are shielded in a lead brick lined enclosure—the robot workcell is itself not enclosed.

The system that required the most extensive modification is currently enclosed in a stainless steel glovebox [5]. This application called for the transfer of samples of Pu-238 oxides into and out of calorimeters for measurement of their heat output. Pu-238 is an intense alpha emitter and, as an oxide, the particulate acquires a charge. These charged particles are very mobile, quickly contaminate any space, and even migrate into conductors shorting them eventually. All drive electronics were removed from the Zymark robot base and wrist, coatings were removed, and all plastic components were replaced with metal. The only components remaining with the robot arm are the servo motors and feedback potentiometers. Remoted electronics were placed in a separate housing and cabled to through the wall of the glovebox using special hermetically sealed feedthrough connectors.

Our experience with radiation environments, gloveboxes, and existing laboratories have led us to begin design of our own robotic arm. The system will be of a gantry geometry and be modular in the *x* and *y* dimensions in increments of 6 inches. This will allow us to size the robot to the existing work space and the intended application. The *z*-axis will be telescoping in on itself to limit the overall height of the robot. The gantry design permits maximum use of the bench space or glovebox floor for modules, while the robot itself uses previously unused space overhead. Laboratory remodelling costs will thus be circumvented. Additional specifications have been reviewed by many researchers and address such areas as material compatibility, precision, controller architecture, tool changing, etc. The arm will be compatible with other commercially available laboratory robotic modules (i.e., syringe stations, balances, centrifuges, etc.). We anticipate having prototypes available within 2 years.
